# Biological Nitrogen Removal Database: A Manually Curated Data Resource

**DOI:** 10.3390/microorganisms10020431

**Published:** 2022-02-12

**Authors:** Tanyaradzwa R. Ngara, Peiji Zeng, Houjin Zhang

**Affiliations:** Department of Biotechnology, College of Life Science and Technology, Huazhong University of Science and Technology, MOE Key Laboratory of Molecular Biophysics, Wuhan 430074, China; rodgersn@hust.edu.cn (T.R.N.); peijizeng@gmail.com (P.Z.)

**Keywords:** biological nitrogen removal, bioreactor systems, microbial communities, wastewater treatment plants, data resource

## Abstract

Biological nitrogen removal (BNR) technologies are the most effective approaches for the remediation of environmental nitrogen pollutants from wastewater treatment plants (WWTPs). Presently, research is going on to elucidate the structure and function of BNR microbial communities and optimizing BNR treatment systems to enhance nitrogen removal efficiency. The literature on BNR microbial communities and experimental datasets is not unified across various repositories, while a uniform resource for the collection, annotation, and structuring of these BNR datasets is still unavailable. Herein, we present the Biological Nitrogen Removal Database (BNRdb), an integrated resource containing various manually curated BNR-related data. At present, BNRdb contains 23,308 microbial strains, 46 gene families, 24 enzymes, 18 reactions, 301 BNR treatment datasets, 860 BNR-associated next-generation sequencing datasets, and 6 common BNR bioreactor systems. BNRdb provides a user-friendly interface enabling interactive data browsing. To our knowledge, BNRdb is the first BNR data resource that systematically integrates BNR data from archaeal, bacterial, and fungal communities. We believe that BNRdb will contribute to a better understanding of BNR process and nitrogen bioremediation research.

## 1. Introduction

In the past few decades’ nitrogen has become a significant contributor to biochemical pollution and the contamination of water sources [1,2]. Biological nitrogen removal (BNR) processes are extensively used to remove nitrogen pollutants in wastewater [3]. BNR widely applied in wastewater treatment plants (WWTPs) includes conventional nitrification and denitrification [3,4]. Recent innovations in the past 30 years have resulted in novel BNR processes being identified, i.e., complete ammonium oxidation (comammox), nitrite/nitrate-dependent anaerobic methane oxidation (n-DAMO), heterotrophic nitrification–aerobic denitrification (HN-AD), anaerobic ammonium oxidation (anammox), and dissimilatory nitrate reduction to ammonium (DNRA), which have emerged as cost- and energy-effective BNR methods [5,6,7,8,9]. Microbial ecology studies characterizing BNR microbial communities, their structure, and the diversity of genes involved in nitrogen bioremediation are essential for determining factors that influence the efficiency and stable long-term operation of BNR systems. The BNR process is improved by developing optimized biotechnological and novel strategies in nitrogen bioremediation [10]. Recently, researchers are making great efforts to apply novel strategies to achieve high nitrogen removal efficiency, for example, coupling anammox with partial-denitrification (PD-A) [11], enhancing the anammox process by switching between warm side-stream and cold mainstream wastewater flows [12], coupling partial nitrification with anammox (PN-A) [13], real-time oxidation reduction potential (ORP) decrease rate control in a PN-A system [14], and achieving simultaneously enhanced nitrogen removal efficiency and electricity generation by coupling bio-electrochemical system (BES) with PD-A [15]. Traditional microbial techniques applied in culturing of BNR microbes in the laboratory have contributed immensely to what is known about the biodiversity of BNR microbial communities in natural and engineered systems. However, these techniques are limited as the majority of microbes are unculturable [16]. The introduction of culture-independent approaches in the late 20th century has helped reveal many important microbial lineages associated with BNR. Culture-independent techniques that are used in microbial ecology studies of BNR microbial communities include 16S rRNA gene amplicon sequencing [17], fluorescent in situ hybridization (FISH) [18], quantitative polymerase chain reaction (qPCR) [19], shotgun metagenomics [20], metagenome-assembled genomes [21] and multi-omics approaches [22].

Advances in relatively cheap and ultra-high throughput next-generation sequencing (NGS) technologies have afforded unprecedented insights into microbial-mediated bioremediation in environmental microbiology studies [23]. Vast quantities of DNA and RNA sequences generated by NGS platforms are deposited into public databases [24]. However, public databases, for example, GenBank [25], UniProtKB [26], and KEGG [27], store a huge amount of biological data. These databases cover other biological aspects apart from BNR processes and hence require foreknowledge of metabolic pathways for accurate usage. The extraction of BNR-related gene datasets from complex public databases is time-consuming. In addition, decades of published scientific literature has produced a vast wealth of data documenting nitrogen bioremediation. BNR-associated datasets are distributed across the literature and public databases, for example, PubMed [25], ScienceDirect [28], and Google Scholar [29], randomly and in an unsystematic manner, making it a challenging task for researchers to extract and comparatively analyze BNR datasets. Recently, the MiDAS field guide database was developed to provide curated taxonomical and physiological information of important microbial communities found in sludge-activated chambers and anaerobic digester systems [30]. However, it only provides taxonomical information based on 16S rRNA gene analyses, and datasets are collected from only 2 types of resources related to wastewater treatment systems. Therefore, it is desirable to develop a knowledge repertoire of manually curated BNR datasets from diverse environmental sources, which contains related information (e.g., functional genes, enzyme functions, and BNR-related datasets) derived from the literature to advance nitrogen bioremediation research. 

Herein, we report the development of a new Biological Nitrogen Removal Database (BNRdb), a manually curated platform which integrates various literature-derived BNR data. To our knowledge, this is the first BNR database that systematically integrates BNR data from archaeal, bacterial, and fungal microbial groups to support BNR functional studies. BNRdb strives to provide a freely accessible and user-friendly platform for not only professional BNR researchers but also the broader scientific community to further investigate the function and activity of BNR microbial communities.

## 2. Methodology

### 2.1. Overview of Database Construction and Content

**Figure 1:**
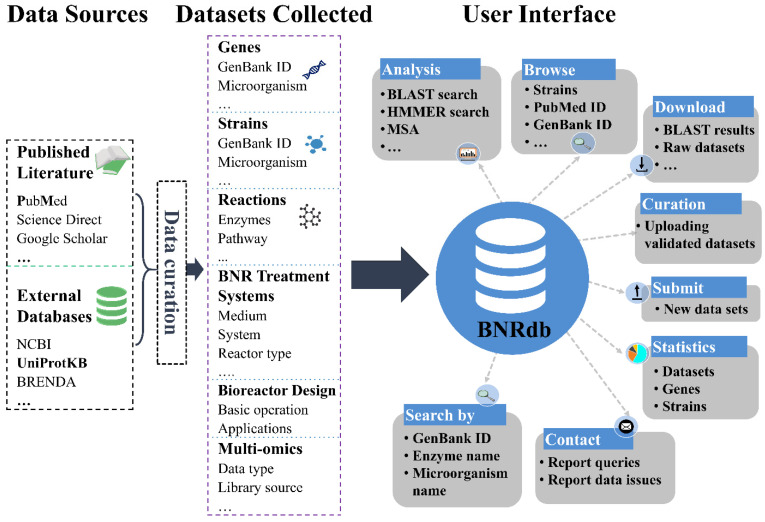
Construction, content, and interface of BNRdb. Data were collected from published literature and databases and curated into six categories. BNRdb provides a user-friendly interface to query, browse, analyze, and download detailed information about these data sets.

The construction of the BNRdb database was based on a review of BNR-related scientific literatures and information extracted from public databases (Figure 1). The Biological Nitrogen Removal Database (BNRdb, freely available at http://bnrdb.genome-mining.cn/) strives (i) to provide easy access to manually curated published data of experimentally validated microbes involved in nitrogen bioremediation identified based on 16S rRNA phylogenetic and functional gene families’ studies; (ii) to gather contextual genomic and literature information for each microbial entry; (iii) to assemble BNR-associated datasets that can be analyzed to improve bioreactor system stability, nitrogen removal efficiencies, and remediation at contaminated sites; and (iv) to provide an intuitive interface to browse, query, visualize, and download BNR information contained in the database.

### 2.2. Data Collection

The criteria established in the collection of the dataset making up the BNRdb were based on the biochemical pathways involved in nitrogen removal: nitrification, comammox, denitrification, anammox, HN-AD, DNRA, and n-DAMO. The following was considered: (i) microbes verified through experiments to possess any one or more of the functional genes encoding enzymes associated with these biochemical pathways; (ii) microbial strains identified from natural or engineered systems that were experimentally validated to have the ability to perform in any one or more of the biochemical pathways in nitrogen removal; and (iii) set experimental parameters and results obtained from experimentally validated studies into BNR in natural and engineered systems. Data collected in the BNRdb database was obtained with these criteria and extensive literature searches in publicly accessible databases such as PubMed [25], Google Scholar [29], and Science Direct [28]. Searches for BNR-related literature were performed using keywords generated based on ‘BNR: nitrification, comammox, denitrification, anammox, HN-AD, DNRA, and n-DAMO’, looking for functional genes, enzymes, and biological treatment systems associated with these biochemical pathways. In an effort to generate more focused search results, the following Boolean search string was used: (‘nitrifiers’ OR ‘complete nitrifiers’ OR ‘denitrifiers’ OR ‘anammox bacteria’ OR ‘HN-AD bacteria’ OR ‘DNRA’ OR ‘n-DAMO’) for the fields [Title, Abstract, Keywords]. The search result returned over 10,000 indexed citations. De-duplication was performed, eliminating duplicate records of literature. Manual curation of the abstracts was performed to eliminate the non-related hits. The experimentally supported BNR events were then manually curated from this scientific literature by at least two researchers. BNR datasets in our database were established by functional validation via a systematic literature review of 1541 peer-reviewed articles. In the BNRdb database, the NCBI GenBank ID accession numbers of the organisms were used as the unique identifiers. However, there is a possibility that some BNR-related datasets (sequences and operational details) were unintentionally omitted. An interactive feature is incorporated in BNRdb, via which contributors can submit those missing BNR datasets and subsequently upload them in future database updates. 

The functional annotations of entries in BNRdb were performed with NCBI GenBank [25], UniProt [26], NCBI Protein [25], KEGG [27], and BRENDA [31]. 

### 2.3. Data Management and Layout

For clarity, functional data associated with the seven biochemical processes involved in BNR was manually curated into six core categories: Genes, Strains, Reactions, Biological treatment systems, Metagenomics, and Bioreactor systems. Internal links were used to connect related datasets on one section of the website to a different section of the same website. For example, the genes section contains curated lists of microbial isolates from which the genes are extracted. For every distinct microbial isolate, their functional data were collected; each microorganism page comprises the reaction stage, encoding gene, protein name, GenBank, and UniProt accession numbers from NCBI [25], UniProtKB [26], external links to the cDNA/protein sequences, environmental source, and scientific references. Information, such as references to literature sources and other external references such as enzyme commission (EC) numbers, is provided as an external link facilitating access to the relevant online resources, extending and supplementing information in the database. 

The strain section contains lists of microbial strains capable of nitrogen removal as indicated by the literature. The information about each entry includes the name of the strain, source of isolation, type of respiration, NCBI GenBank ID, substrates utilized, electron donors, electrons acceptors, length of the cDNA sequence, and external link to the scientific references. The annotation fields used for microbial strain entries in BNRdb are described (Supplementary Tables S1 and S2).

The reaction section displays the literature-derived information of the reactions associated with the biochemical processes of nitrogen removal: nitrification, denitrification, anammox, comammox, DNRA, and n-DAMO. Extracted information relevant to enzymes associated with the BNR biochemical processes from the BRENDA database. Each enzyme entry contains information such as enzyme name, gene cluster, EC number, KEGG orthology IDs, classification, substrates, products, genes, brief detail on the subunits that make up the functional enzymes, and scientific references. Peer-reviewed literature extracted from PubMed that studied the biochemistry, function, and mechanism of the enzymes is provided as external links. This section also contains pathway maps, which users can access the outlines of how the nitrification, denitrification, anammox, comammox, DNRA, and n-DAMO reactions proceed. The reaction pathways display the series of reactions involved, the enzymes at every stage in the reaction pathway, their EC numbers, the genes encoding the enzymes, reactants, and products of the BNR processes.

The BNR treatment systems section displays relevant BNR experimental conditions and datasets in the removal of nitrogen from natural wetlands and engineered systems. Each entry contains various information extracted from literature: the influent source, type of system, bioreactor, bio-carrier medium, respiration, electron donor, electron acceptor, loading and removal rates of ammonium and nitrate, nitrogen removal efficiencies, and scientific references. Entries in this section were each assigned unique IDs for linking the data to external databases. The metagenomics section displays BNR-related multi-omics sequencing datasets of entire microbial communities isolated from diverse environments. Each entry contains information about the data type, library source, external links to the Sequence Read Archive database, and references to the published literature. The bioreactor section displays information about the types of bioreactors used in BNR in wastewater treatment systems. Information on the basic operation of commonly used biofilm carriers, variations of bioreactors developed based on the main types, the benefits, and limitations of each bioreactor system were collected. A general description of modification applied to the reactors for BNR in large scale community applications is also included.

The inter-connecting nature of BNRdb affords users the ease of navigating through the website, facilitating access to all the data within the database irrespective of the starting point.

### 2.4. Database Architecture and Web Interface Implementation

The backend framework was implemented in Django 2.0 using Python 3.6. The front-end framework was implemented using HTML5, CSS3, and JavaScript with Bootstrap 4. Data were stored and managed in MariaDB 5.5.56. Protein and nucleotide alignment were implemented with HMMER 3.2 [32] and BLAST [33], respectively. Multiple sequence alignment and phylogenetic tree generation were implemented with Clustal Omega [34] and PHYLIP [35] packages. 

## 3. Results 

### 3.1. Data Statistics

**Figure 2:**
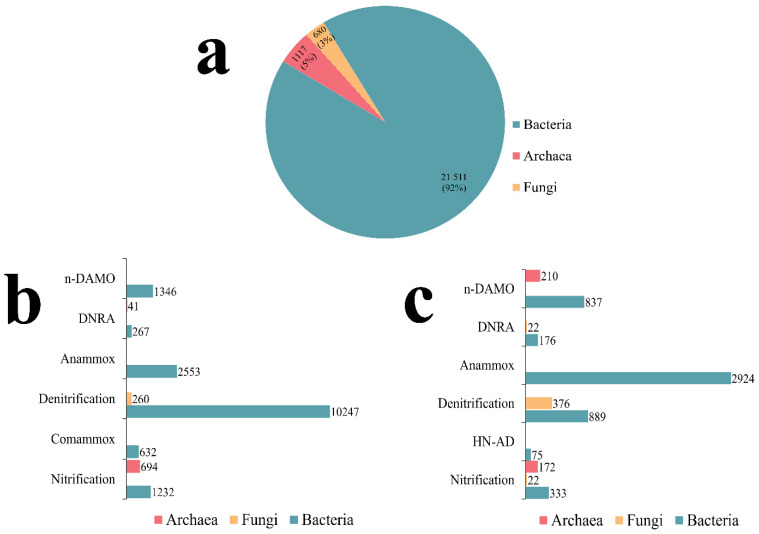
Statistics of nitrogen-removing microbes in BNRdb. (**a**) The distribution of microbial strains (see in Table S3) in BNRdb. (**b**) Distribution of microbial strains identified based on functional gene analysis. (**c**) Distribution of microbial strains identified based on either 16S rRNA gene analysis or functional screening studies.

The current version of BNRdb contains information on 23,308 microbial strains, 46 gene families, 24 enzymes, 18 reactions, 301 BNR treatment systems datasets, 860 BNR-associated next-generation sequencing datasets, and 6 commonly bioreactors in BNR from wastewater plants and other environmental systems. There are 21,511 microbial strains for bacteria, 1117 for archaea, and 680 for fungi (Figure 2a). Of these, 75.67% (16,277 strains), 65.80% (735 strains), and 38.24% (260 strains) of the bacterial, archaeal, and fungal strains, respectively, were identified based on functional gene analysis (Figure 2b). 

A total of 46 gene families were found through analyzing 6 BNR processes in the nitrogen cycling, namely, nitrification, comammox, denitrification, anammox, DNRA, and n-DAMO.

Nitrification: Sequences were collected from eight functional genes encoding the nitrification enzymes: *amoA*, *amoB*, *amoC*, *hao*, *nxrA*, *nxrB*, *nxrC*, and *nxrD*. The *amoCAB* operon is responsible for NH_4_^+^ oxidation to NH_2_OH, the *hao* gene is responsible for conversion of NH_2_OH to NO, and *nxrABCD* is responsible for NO_2_^−^ oxidation into NO_3_^−^. The prokaryotic domains, Archaea and Bacteria, were regarded as two separate groups for the retrieval of sequences of *amoA*, *amoB*, and *amoC* gene families. A total of 694 and 1232 archaeal and bacterial sequences were collected, respectively. The *amoA* gene was the most prevalent in both archaeal and bacterial groups, accounting for 87.61% and 60.31% of the total entries, respectively.

Comammox: Comammox *Nitrospira* sequences were collected based on the *amoA* functional gene, which encodes the *AmoA* subunit of the ammonia monooxygenase enzyme. There are two distinct clades (clade A and B) of comammox *Nitrospira* formed by different *amoA* orthologs genes from comammox *Nitrospira* bacteria. A total of 632 bacterial sequences were collected.

Denitrification: Bacterial sequences were retrieved from 17 gene families: *narG*, *narH*, *narJ*, *narI*, *narZ*, *narY*, *narV*, *narW*, *napA*, *napB*, *napC*, *napD*, *nirK*, *nirS*, *norB*, *norC*, and *nosZ*. The genes in clusters *napEDABC*, *narGHJI*, and *narZYVW* were handled as individual entities. During the denitrification, *narGHJI*, *narZYVW*, and *napEDABC* gene clusters mediate the reduction of NO_3_^−^ to NO_2_^−^, *nirK* and *nirS* genes are associated with the reduction of NO_2_^−^ to NO, *norCB* genes are responsible for the reduction of NO to N_2_O, and the *nosZ* genes are associated with the reduction of N_2_O to N_2_. The BNRdb contains 10,247 denitrifier sequences, with the majority being the *nirK* and *nirS* genes sequences, encoding the copper and cytochrome cd1 type forms of the metalloenzyme nitrite reductase. The genes encoding the nitrite reductase account for 86.69% of the database’s bacterial denitrification genes.

Fungal denitrification: Fungal sequences of two gene families, *nirK* and *p450nor*, were retrieved. The *nirK* and *p450nor* genes are genes associated with reducing NO_2_^−^ to NO and NO to N_2_O, respectively. A total of 260 fungal sequences were collected. 

Anammox: Anammox bacterial sequences were collected from eight gene families: *hzsA*, *hzsB*, *hzsC*, *hzo*, *hdh*, *ccsA*, *ccsB*, and *ccsX*. The *hzsCBA* gene cluster is responsible for coupling NH_4_^+^ and NO producing hydrazine (N_2_H_4_), and the *hzo* and *hdh* genes are associated with N_2_H_4_ oxidation to N_2_. The *ccsA*, *ccsB*, and *ccsX* gene families included in this database are of interest to the scientific community as they are key to the unique anammox metabolism. The BNRdb database contains a total of 2553 anammox bacteria sequences. The *hzsCBA* gene cluster accounts for 48.69% of the BNRdb’s anammox gene sequences. 

DNRA: Sequences were collected from seven functional genes encoding the enzymes associated with the DNRA process: *nrfA*, *nrfB*, *nrfC*, *nrfD*, *nrfH*, *nirB*, and *nirD*. The *nrfABCDEFG* and *nirBD* gene clusters are associated with the reduction of NO_2_^−^ to NH_4_^+^ without any nitrogenous intermediates being produced. A total of 267 bacterial sequences were collected.

n-DAMO: Sequences were collected from three functional genes encoding the n-DAMO enzymes: *mcrA*, *nod*, and *pmoA*. The *mcrABG* gene cluster is associated with the anaerobic metabolism of methane, the *pmoCAB* gene cluster is associated with methane oxidation to methanol, and the *nod* gene is responsible for the direct disproportion of NO into N_2_ and O_2_. A total of 41 and 1346 archaeal and bacterial sequences were collected, respectively.

BNRdb has 6036 microbial strains collected from the literature based on 16S and 18S rRNA gene analysis (see https://github.com/monsterZeng/BNRdb). Of the total microbial strains (Figure 2a), the bacterial, archaeal, and fungal strains account for 25.32% (5234 strains), 34.20% (382 strains), and 61.76% (420 strains), respectively (Figure 2c). Of the nitrifiers collection in BNRdb, aerobic ammonia-oxidizing archaea (AOA) belong to 4 phyla and 2 genera, and aerobic ammonia-oxidizing bacteria (AOB) belong to 8 phyla and 33 genera. Nitrifying bacteria are separated into AOB and nitrite-oxidizing bacteria (NOB), as this separation is essential for understanding the ecosystem. Fungal heterotrophic nitrifiers in the BNRdb database belong to two phyla and five genera. Bacterial denitrifiers in BNRdb belong to 6 phyla and 126 genera, whilst fungal denitrifiers belong to 4 phyla and 66 genera. The anammox bacteria recorded in the database belong to the phylum *Planctomycetes* and six genera (see https://github.com/monsterZeng/BNRdb). Until now, anammox bacteria are found only in six *Candidatus* genera [36]. In BNRdb, entries for bacteria capable of HN-AD belong to 4 phyla and 25 genera; bacteria capable of DNRA belong to 5 phyla and 33 genera, whilst we recorded one *Aspergillus terreus* fungal entry capable of DNRA and n-DAMO bacterial entries belong to the phylum NC10 and genus *Candidatus Methylomirabilis* whilst the n-DAMO archaeal entries in BNRdb belong to the phylum *Euryarchaeota* and genus *Candidatus Methanoperedens*. At present, the coupling of the anaerobic oxidation of methane to nitrate appears to be a distinctive trait associated with the bacterial genus Ca. *Methylomirabilis* and the archaeal genus *Candidatus Methanoperedens* [37]. The database also currently contains 860 BNR-associated next-generation sequencing datasets.

### 3.2. Search Tools

The BNRdb database (http://bnrdb.genome-mining.cn/) provides an intuitive interface to browse, query, and visualize extensive information about BNR microbial strains. The search bar on each webpage provides an interface for searching the annotated experimental datasets in the database using gene symbols, gene alias, gene, and protein sequence IDs. The user can choose filtering options based on the keyword, rendering the search more specific. 

A set of bioinformatic utilities for the characterization and taxonomic placement of novel BNR associated microbial strains were also included. Users can use the BLAST and HMMER alignment modules implemented in BNRdb to query the database. Users can submit either their nucleotide or protein bacterial query sequence in FASTA format in the BLAST and HMMER search tools, respectively, to detect nucleotide or protein sequence homologs from the manually curated, high-quality sequences in the BNRdb. The Clustal O alignment module implemented in BNRdb allows users to perform multiple sequence alignments, which reveal subtle similarities between sequences. Phylotree.js was implemented to explore an interactive analysis of the phylogeny of BNR microbial strains. This platform facilitates the interactive probing and manipulation of the phylogenetic tree, such as selecting nodes and swapping branches.

### 3.3. Usage and Functionalities

The database provides example sequences to guide users on using the analytical tools and performing bioinformatic analysis. 

**Figure 3:**
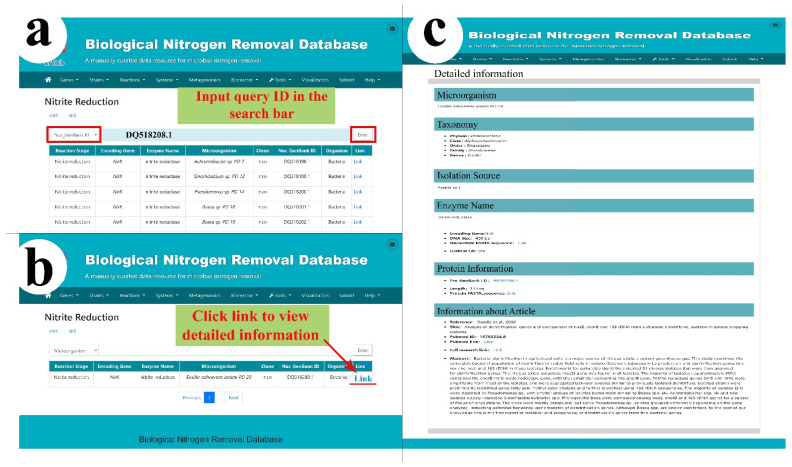
Overview of the BNRdb user interface to access data. (**a**) Users can input the GenBank ID accession number for querying and filtering. For example, accession “DQ518208.1”. (**b**) The returned search result can be further explored by clicking the link to access the detailed information. (**c**) The comprehensive information of the denitrifying microbial strain.

For example, selecting the “Nuc_GenBankID” filter in the search bar and inputting NCBI GenBank accession number “DQ518208.1” as the keyword (Figure 3a), the query result is displayed, with the features of reaction stage, encoding genes, enzyme names, microorganisms, accession IDs, references, and a link for detailed information (Figure 3b). By clicking the “Link” tab, the detailed information for the denitrifying isolate is shown (Figure 3c). The information presented on this page comprises the microorganism’s taxonomy, isolation source, gene information, protein information, and information about the article. The gene nucleotide sequences, protein sequences, and references to the research articles are also provided as external links.

The online BLAST and HMMER interfaces can be used to input the bacterial query sequence(s) and search against all the manually curated high-quality nucleotide or protein sequences in our database (Supplementary Figure S1a,b). Users have the option to download their sequence homologs results report for further analysis. The BLAST and HMMER reports consist of three main components: the header comprises text indicating the nature of the query sequence and the target sequence database. There is a section for one-line descriptions of scores for sequences matching the query sequence for a quick overview of browsing. The third component is for domain annotations for each of the database sequence alignment matches compared to the query sequence. For the first hit in the list, the accession number is DQ420239.1; the definition is *Pseudomonas grimontii* isolate PD 9 nitric oxide reductase; the score is 484; and the E-value is 2 × 10^−137^. All of the hits in this BLAST report have very low E-values (much less than 1), meaning that these alignments are more precise and truly meaningful (Supplementary Figure S2).

Users can construct the phylogenetic tree diagrams showing evolutionary relationships between their query sequences and those sequences found in the BNRdb database (Supplementary Figure S1c,d). The example sequences were used to infer the phylogenetic tree of denitrifying isolates that possess the nitric oxide reductase encoding gene (Supplementary Figure S3). The Clustal Omega package with 1000 cycles of iterative refinement facilitated the alignment of the protein sequences. Clustal O uses seeded guide trees and hidden Markov models (HMMs) profile-profile techniques to generate multiple alignments [34]. Simple phylogeny provides rapid phylogenetic tree reconstruction using the Approximate Maximum Likelihood method of PHYLIP from the choice of either nucleotide or protein alignment [38]. The tree is displayed in Phylotree.js, a JavaScript library for working with phylogenetic trees [35].

The BNRdb database presents experimental datasets of previous and current studies into BNR treatment systems from lab scale to full-scale applications. Several parameters presented in this section, such as feed nitrate, nitrogen loading rate, carbon, and energy source, nitrogen removal rate, and nitrogen removal efficiencies, are instrumental in helping scientists determine the appropriate wastewater treatment technology and bioreactor technology and configuration (Supplementary Figure S4). 

In the BNRdb database, the six commonly used bioreactors configurations for maintaining BNR microbes are included. Users can access information on the basic operation, biofilm carriers used, variations of the bioreactors, and the benefits and limitations associated with each of these bioreactor configurations, which is helpful for formulating an effective nitrogen removal strategy for wastewater treatment and improving the cultivation of slow-growing BNR microbes (Supplementary Figure S5). 

### 3.4. Technical Validation and Updating of the Database

Submission of data—An interactive data submission feature is incorporated into the BNRdb to generate clean and uniform data sets that can be useful to other users (Supplementary Figure S6). Contributors who wish to submit new data sets related to BNR, which are not already included in the database, can use this feature following the submission guidelines. The administrator/curator may then contact the authors who submitted a new dataset to be the editor of that particular dataset and engage in reviewing any inaccurate or potentially missing data during the validation stage. The curator of the database will then upload the new dataset after its validation.

Download page—A download page is provided, enabling users to download the gene sequences, strains information, BNR treatment systems, and bioreactor data tables. By properly citing the original work, users are allowed to redistribute and reproduce in any form. 

Database update—BNRdb is in its release 1.0 and will continually be updated to accommodate new literature entries that are continuously updated on the NCBI and other public repositories. Manual curation of data will be carried out to detect and remove multiple data entries when conflicting datasets are detected.

Users’ feedback—Any queries or data issues concerning the BNRdb can be reported via the contact feature incorporated in the BNRdb. The administrator will respond to their queries.

## 4. Discussion

BNR microbial communities and BNR treatment experimental datasets are important for the optimization of conventional WWTPs and the development of novel BNR treatment technologies in order to meet the stringent discharging standards. To achieve this purpose, information sharing is essential for the development of BNR research. We developed BNRdb, a database for exploring the various BNR-related data of BNR microbial communities manually curated from published literature. 

The BNRdb database incorporates BNR-associated genes/strains, BNR treatment experimental datasets and metagenomic datasets from BNR engineered systems and diverse environments. Natural environments such as intertidal zones, where the oxic/anoxic conditions vary through time, are valuable microbial sources for discovering novel BNR metabolic pathways and microbial functions [39,40]. Users can thus access the BNR information from natural environments held within the BNRdb to evaluate the real applicability of novel BNR microbial strains in the optimization of BNR treatment systems. Understanding the syntrophic interactions that occur within microbial communities and dynamics in BNR treatment systems is crucial for the efficient and stable removal of nitrogen from WWTPs and extending knowledge of microbe-driven biogeochemical cycles [22,41]. Therefore, literature-derived (meta-) genomic and multi-omics datasets within the BNRdb are helpful to promote research to gain new insights into the metabolic functions of BNR microbial communities. The elucidation of novel metabolic pathways will flourish the development of simpler, cheaper, and greener biological wastewater treatment technologies [42]. Achieving efficient BNR treatment system performance is determined by process control and optimizing environmental conditions to ensure optimal functional diversity within the BNR treatment system [43]. BNRdb provides summary information about BNR experimental datasets and system operational conditions. Users can thus perform comparative analysis studies with the experimental datasets held within BNRdb in order to determine the optimal conditions for enhancing the survival and performance of BNR microbial communities in the removal of nitrogen from WWTPs. 

## 5. Conclusions and Future Directions

This study presents the BNRdb, a publicly available resource entirely dedicated to BNR. The BNRdb integrates data extracted from publicly accessible data repositories and scientific literature to provide insightful information about BNR, which would foster studies of nitrogen removal microbial communities and the development of technologies for the efficient removal of nitrogen pollutants. It is expected that the BNRdb database will be proven to be a valuable resource for professional BNR researchers and the broader scientific community working to advance our understanding of BNR systems.

With advances in NGS technologies developing exponentially, the number of multi-omics approaches towards the functional analysis of BNR communities is expected to keep increasing. Hence, efforts will be made to periodically update the BNRdb database with the intention of including the most recent BNR related research. A future step in the development of BNRdb will be dedicated to improving the information available in BNRdb, such as collecting detailed experimental details, cataloging BNR-related function genes, and incorporating web-accessible bioinformatics tool for analyzing and visualizing BNR-related high throughput NGS data. We plan to integrate 16S and functional gene information with experimental data. We believe that this correlation function can offer valuable insights into how the inherent genes or strains contribute to the BNR efficiency. We will look to construct an additive database on syntrophic microbes which contribute to the BNR processes. This will help in extending our knowledge of syntrophic associations of the microbial community in WWTPs, electron transfer modes of microbes, and understanding the ecosystem function within BNR treatment systems.

## Data Availability

The datasets associated with this article are available in the Github repository (https://github.com/monsterZeng/BNRdb).

## Supplementary Materials

The following supporting information can be downloaded at: www.mdpi.com/article/10.3390/microorganisms10020431/s1, Figure S1. Overview of the BNRdb user-interface to access data.; Figure S2. Description of fields of the BLAST hits on the query sequence.; Figure S3. The phylogenetic tree obtained in PHYLIP for microbial denitrifiers that possess the nitric oxide reductase gene using the example sequences provided as references to use in BNRdb.; Figure S4. Screenshot of the BNR treatment systems experimental methods and conditions.; Figure S5. Screenshot display of the bioreactor display section using Fluidized Bed Reactor as an example.; Figure S6. Screenshot of the submission page.; Table S1. Description of entry fields used to annotate BNR microbes based on functional genes that encode functional enzymes involved in BNR processes.; Table S2. Description of fields used to annotate nitrogen-removing microbial strains based on 16S rRNA gene analysis.; Table S3. The distribution of microbial strains in BNRdb database.
