# Supplementary material for: Biological Nitrogen Removal Database: A Manually Curated Data Resource
Source: Microorganisms. 2022 Feb 12;10(2):431. doi: 10.3390/microorganisms10020431 (PMC8874995; doi:10.3390/microorganisms10020431)
Supplement: Supplementary file 1 [file microorganisms-10-00431-s001.zip › microorganisms-1565773-supplementary/Supplementary_Info/Supplementary materials.pdf]

Supplementary materials

Journal: *Microorganisms*

Title: BNRdb: A Manually Curated Data Resource for Biological Nitrogen Removal

Tanyaradzwa R. Ngara[1,#], Peiji Zeng[1,#] and Houjin Zhang[1,*]

[1] *Department of Biotechnology, College of Life Science and Technology, Huazhong University of Science and Technology, MOE Key Laboratory of Molecular Biophysics, Wuhan 430074, China.*

* Corresponding author: Dr. Houjin Zhang,

Address: College of Life Science and Technology, Huazhong University of Science and Technology, MOE Key Laboratory of Molecular Biophysics, Wuhan 430074, China.

Email address: hjzhang@hust.edu.cn

Telephone: +86 27 87793085

[#]These authors contributed equally.

Annotation Procedure of Nitrogen-removing Microbes

Table S1. Description of entry fields used to annotate BNR microbes based on functional genes that encode functional enzymes involved in BNR processes.

Field Name	Description
GenBank ID	Unique database identifier for a particular key gene
Microorganism	Microorganism that possess the key gene
Isolation Source	The geographical source of the organism from which the sequence was derived
Taxonomy	Identification and Classification of the microorganism
Encoding Gene	Approved name of the gene
Enzyme Name	Approved common name of the protein encoded for by the gene
DNA size(bp)	Size of the DNA of the gene
Nucleotide FASTA sequence	External link to NCBI to access the nucleotide sequence of the gene in FASTA format
UniProtKB ID	External link to UniProtKB database
Protein GenBank IB	External link to the NCBI protein sequence
Length (aa)	Number of amino acids in the canonical sequence
Protein FASTA sequence	External link to the NCBI amino acid sequence of the gene in FASTA format
Reference	Name of authors of the research article
Title	The name of the academic paper that summarizes the main ideas of the study
PMID	External link to PubMed
Research Link	External link to full article
Abstract	Quick overview of the academic paper

Table S2. Description of fields used to annotate nitrogen-removing microbial strains based on 16S rRNA gene analysis.

Field Name	Description
GenBank ID	Unique database identifier for BNR microbe identified by their ability to be involved in any of the BNR processes
Microorganism	Microorganism that has the ability to perform BNR
Isolation Source	The geographical source from which the microorganism was taken
Taxonomy	Identification and Classification of the microorganism
Electron Donor/ Energy Source	A reactant that donates electrons in an oxidation-reaction in an oxidation-reduction reaction
Final product	The intermediary and final end products of the BNR process under study
Respiration	Ability of the BNR microbe to perform BNR proccesses in anaerobic or aerobic conditions
Electron Acceptor	The nitrogenous compound/ intermediate consumed or reduced
Reference	Name of authors of the research article
Title	The name of the academic paper that summarizes the main ideas of the study
PMID	External link to PubMed
Research Link	External link to full article
Abstract	Quick overview of the academic paper

Explore and browse interface

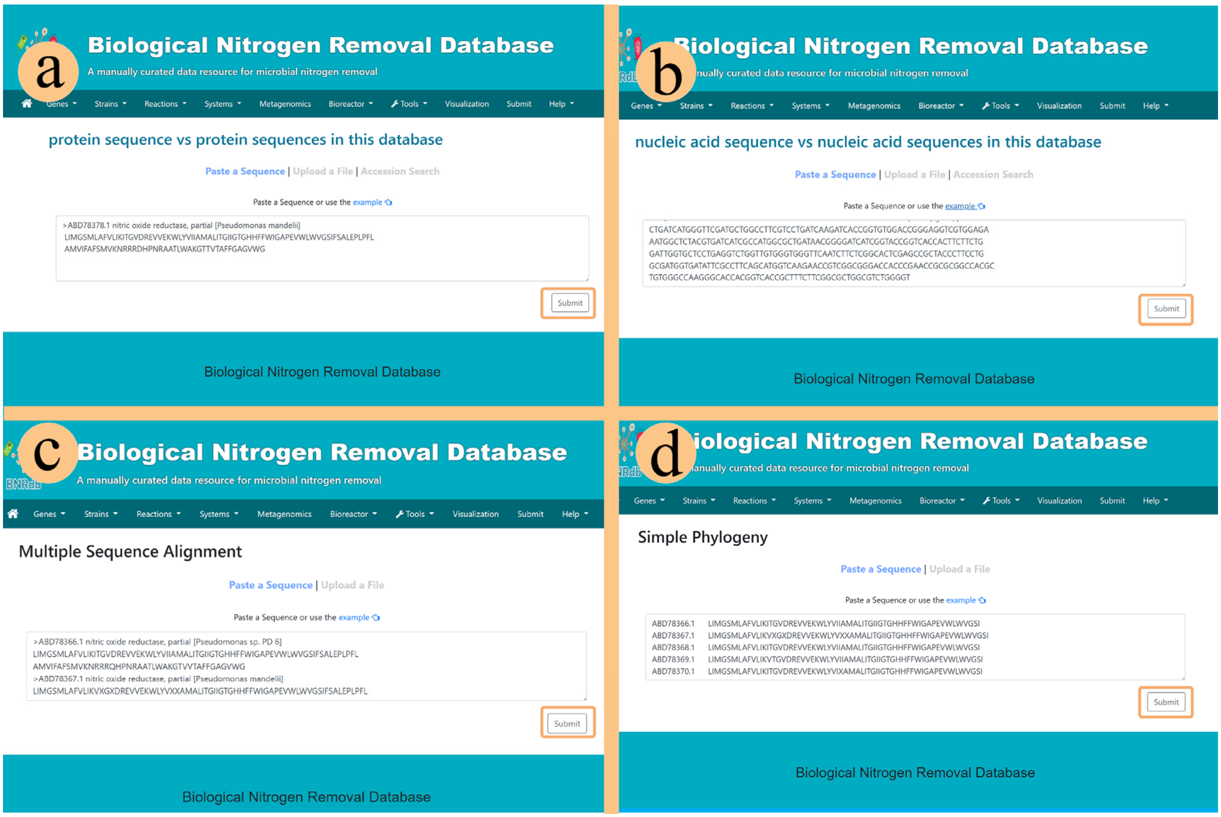

Figure S1. Overview of the BNRdb user-interface to access data. (A) BLAST search and (B) HMMER Users can perform local similarity searches between nucleotide and protein sequences, respectively. (C) Multiple sequence alignment using Clustal O. (D) User can use the simple phylogeny to infer homology and the evolutionary relationship between the sequences studies by submitting their aligned sequences either by pasting it in or uploading the file with the aligned sequences.

BLAST search results

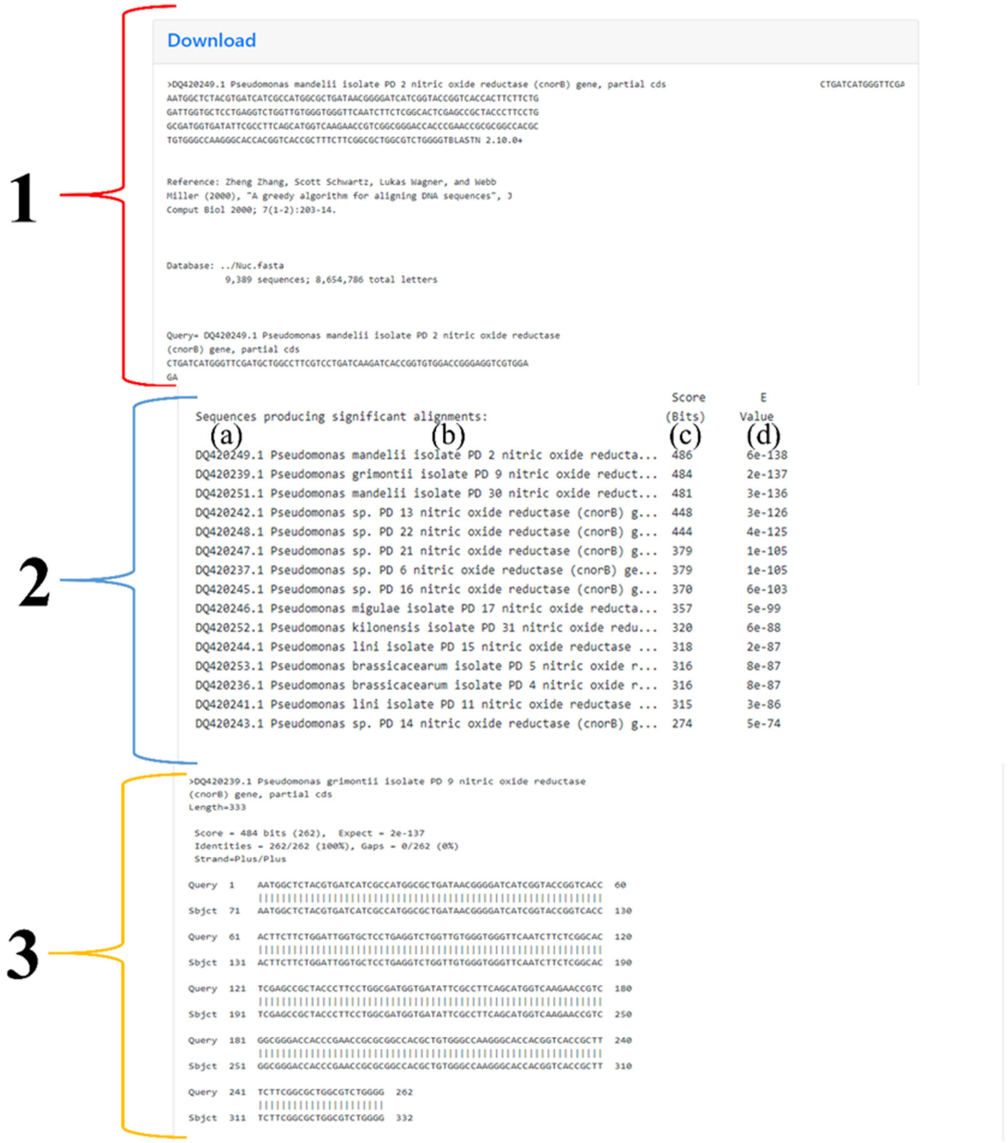

Figure S2. Description of fields of the BLAST hits on the query sequence. (1) the header of the BLAST report: the top line shows information about the query sequence. The scientific article that describes BLAST is then cited. Followed by the summary of the database searched. The length of the query sequence is also shown. (2) the one-line description in the BLAST report: Each line has four fields (a) accession number; (b) description of sequence; (c) the score of the alignment in bits. Hits found at the top of the list have a higher score; (d) with the E-value providing an estimate of statistical significance. (3) Pairwise sequence alignment from the BLAST report. The alignment is

preceded by the accession number, description line and the length of the matched sequence in nucleotides. This is followed by the bit score (with the raw score shown in parenthesis) and the E-value. Next comes the line which shows information concerning the number of identical residues in the pairwise alignment (identities) and the number of gaps the alignment has if any. Lastly, the actual alignment is displayed, with the query sequence on top and the database sequence match below (which labelled Sbjct). The numbers on the left and right help show the position in the nucleotide sequence. Note that (-) indicates deletions and insertions within the sequence. Similarities between the sequences are indicated by a line between the two sequences. If the query and subject sequence do not have similar nucleotide bases at a given point the area between the two sequence is presented as blank.

The phylogenetic tree obtained in Phylotree.js

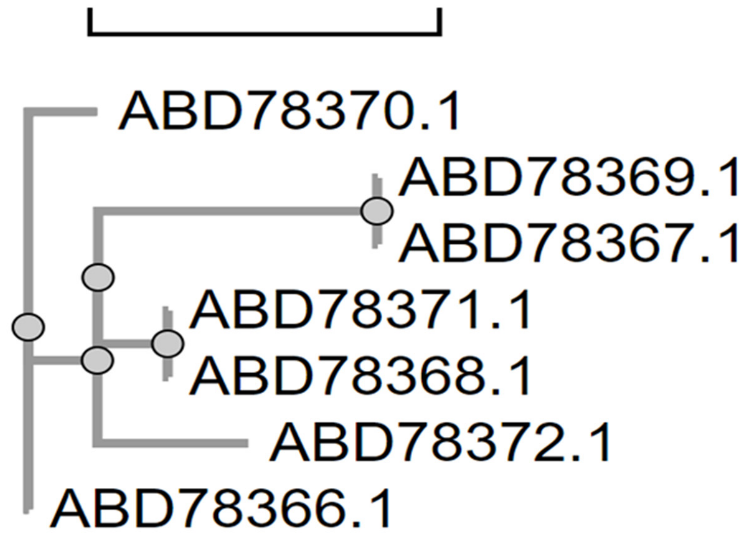

Figure S3. The phylogenetic tree obtained in PHYLIP for microbial denitrifiers that possess the nitric oxide reductase gene using the example sequences provided as references to use in BNRdb.

BNR Treatment Systems page

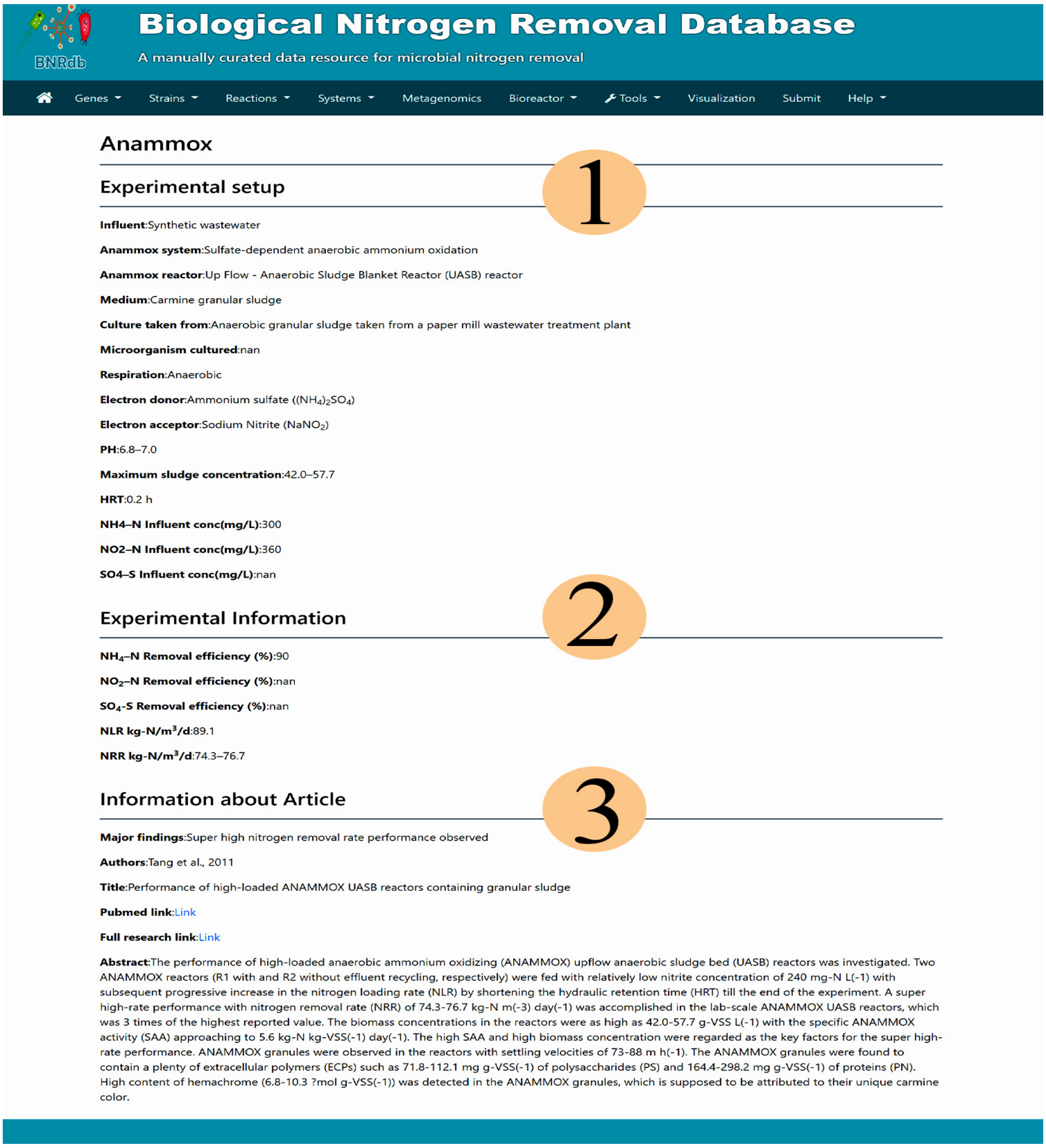

Figure S4. Screenshot of the BNR treatment systems experimental methods and conditions. (1) Experimental setup. (2) Experimental results in nitrogen removal from the system. (3) Literature information is provided with links to external databases.

Bioreactor Page

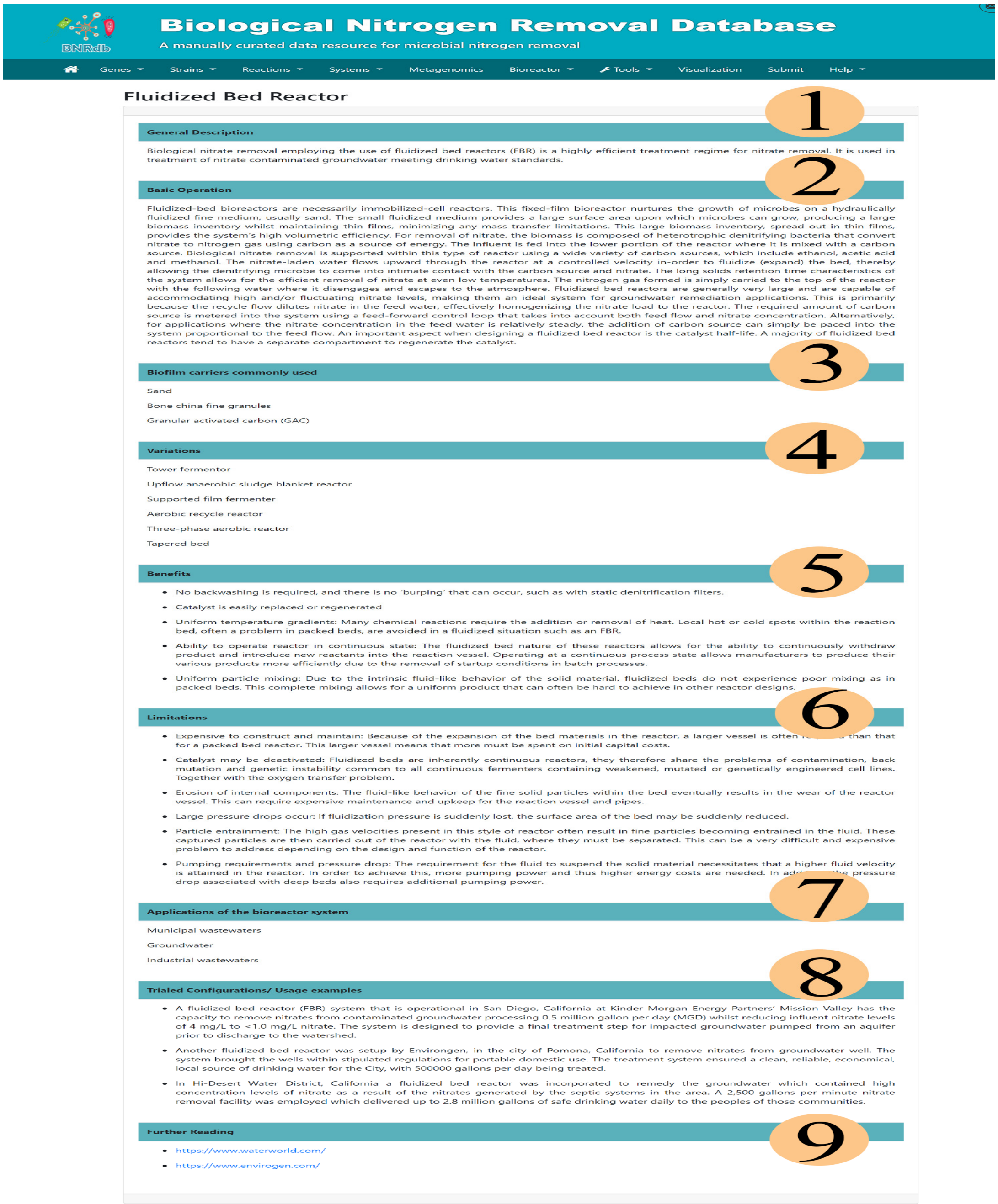

Biological Nitrogen Removal Database

A manually curated data resource for microbial nitrogen removal

BNRdb

Genes | Strains | Reactions | Systems | Metagenomics | Bioreactor | Tools | Visualization | Submit | Help

Fluidized Bed Reactor

General Description

Biological nitrate removal employing the use of fluidized bed reactors (FBR) is a highly efficient treatment regime for nitrate removal. It is used in treatment of nitrate contaminated groundwater meeting drinking water standards.

Basic Operation

Fluidized-bed bioreactors are necessarily immobilized-cell reactors. This fixed-film bioreactor nurtures the growth of microbes on a hydraulically fluidized fine medium, usually sand. The small fluidized medium provides a large surface area upon which microbes can grow, producing a large biomass inventory whilst maintaining thin films, minimizing any mass transfer limitations. This large biomass inventory, spread out in thin films, provides the system's high volumetric efficiency. For removal of nitrate, the biomass is composed of heterotrophic denitrifying bacteria that convert nitrate to nitrogen gas using carbon as a source of energy. The influent is fed into the lower portion of the reactor where it is mixed with a carbon source. Biological nitrate removal is supported within this type of reactor using a wide variety of carbon sources, which include ethanol, acetic acid and methanol. The nitrate-laden water flows upward through the reactor at a controlled velocity in-order to fluidize (expand) the bed, thereby allowing the denitrifying microbe to come into intimate contact with the carbon source and nitrate. The long solids retention time characteristics of the system allows for the efficient removal of nitrate at even low temperatures. The nitrogen gas formed is simply carried to the top of the reactor with the following water where it disengages and escapes to the atmosphere. Fluidized bed reactors are generally very large and are capable of accommodating high and/or fluctuating nitrate levels, making them an ideal system for groundwater remediation applications. This is primarily because the recycle flow dilutes nitrate in the feed water, effectively homogenizing the nitrate load to the reactor. The required amount of carbon source is metered into the system using a feed-forward control loop that takes into account both feed flow and nitrate concentration. Alternatively, for applications where the nitrate concentration in the feed water is relatively steady, the addition of carbon source can simply be paced into the system proportional to the feed flow. An important aspect when designing a fluidized bed reactor is the catalyst half-life. A majority of fluidized bed reactors tend to have a separate compartment to regenerate the catalyst.

Biofilm carriers commonly used

Sand

Bone china fine granules

Granular activated carbon (GAC)

Variations

Tower fermentor

Upflow anaerobic sludge blanket reactor

Supported film fermenter

Aerobic recycle reactor

Three-phase aerobic reactor

Tapered bed

Benefits

- No backwashing is required, and there is no 'burping' that can occur, such as with static denitrification filters.
- Catalyst is easily replaced or regenerated
- Uniform temperature gradients: Many chemical reactions require the addition or removal of heat. Local hot or cold spots within the reaction bed, often a problem in packed beds, are avoided in a fluidized situation such as an FBR.
- Ability to operate reactor in continuous state: The fluidized bed nature of these reactors allows for the ability to continuously withdraw product and introduce new reactants into the reaction vessel. Operating at a continuous process state allows manufacturers to produce their various products more efficiently due to the removal of startup conditions in batch processes.
- Uniform particle mixing: Due to the intrinsic fluid-like behavior of the solid material, fluidized beds do not experience poor mixing as in packed beds. This complete mixing allows for a uniform product that can often be hard to achieve in other reactor designs.

Limitations

- Expensive to construct and maintain: Because of the expansion of the bed materials in the reactor, a larger vessel is often [illegible] than that for a packed bed reactor. This larger vessel means that more must be spent on initial capital costs.
- Catalyst may be deactivated: Fluidized beds are inherently continuous reactors, they therefore share the problems of contamination, back mutation and genetic instability common to all continuous fermenters containing weakened, mutated or genetically engineered cell lines. Together with the oxygen transfer problem.
- Erosion of internal components: The fluid-like behavior of the fine solid particles within the bed eventually results in the wear of the reactor vessel. This can require expensive maintenance and upkeep for the reaction vessel and pipes.
- Large pressure drops occur: If fluidization pressure is suddenly lost, the surface area of the bed may be suddenly reduced.
- Particle entrainment: The high gas velocities present in this style of reactor often result in fine particles becoming entrained in the fluid. These captured particles are then carried out of the reactor with the fluid, where they must be separated. This can be a very difficult and expensive problem to address depending on the design and function of the reactor.
- Pumping requirements and pressure drop: The requirement for the fluid to suspend the solid material necessitates that a higher fluid velocity is attained in the reactor. In order to achieve this, more pumping power and thus higher energy costs are needed. In [illegible] the pressure drop associated with deep beds also requires additional pumping power.

Applications of the bioreactor system

Municipal wastewaters

Groundwater

Industrial wastewaters

Trialed Configurations/ Usage examples

- A fluidized bed reactor (FBR) system that is operational in San Diego, California at Kinder Morgan Energy Partners' Mission Valley has the capacity to remove nitrates from contaminated groundwater processing 0.5 million gallon per day (MGD) whilst reducing influent nitrate levels of 4 mg/L to <1.0 mg/L nitrate. The system is designed to provide a final treatment step for impacted groundwater pumped from an aquifer prior to discharge to the watershed.
- Another fluidized bed reactor was setup by Environgen, in the city of Pomona, California to remove nitrates from groundwater well. The system brought the wells within stipulated regulations for portable domestic use. The treatment system ensured a clean, reliable, economical, local source of drinking water for the City, with 500000 gallons per day being treated.
- In Hi-Desert Water District, California a fluidized bed reactor was incorporated to remedy the groundwater which contained high concentration levels of nitrate as a result of the nitrates generated by the septic systems in the area. A 2,500-gallons per minute nitrate removal facility was employed which delivered up to 2.8 million gallons of safe drinking water daily to the peoples of those communities.

Further Reading

- https://www.waterworld.com/
- https://www.envirogen.com/

Figure S5. Screenshot display of the bioreactor display section using Fluidized Bed Reactor as an example. (1) General description. (2) Basic operation. (3) Carriers commonly used. (4) Variations. (5) Benefits of the reactor system (6), and Limitation associated with its use. (7) Possible applications. (8) Trailed configurations. (9) Further reading.

Submissions Page

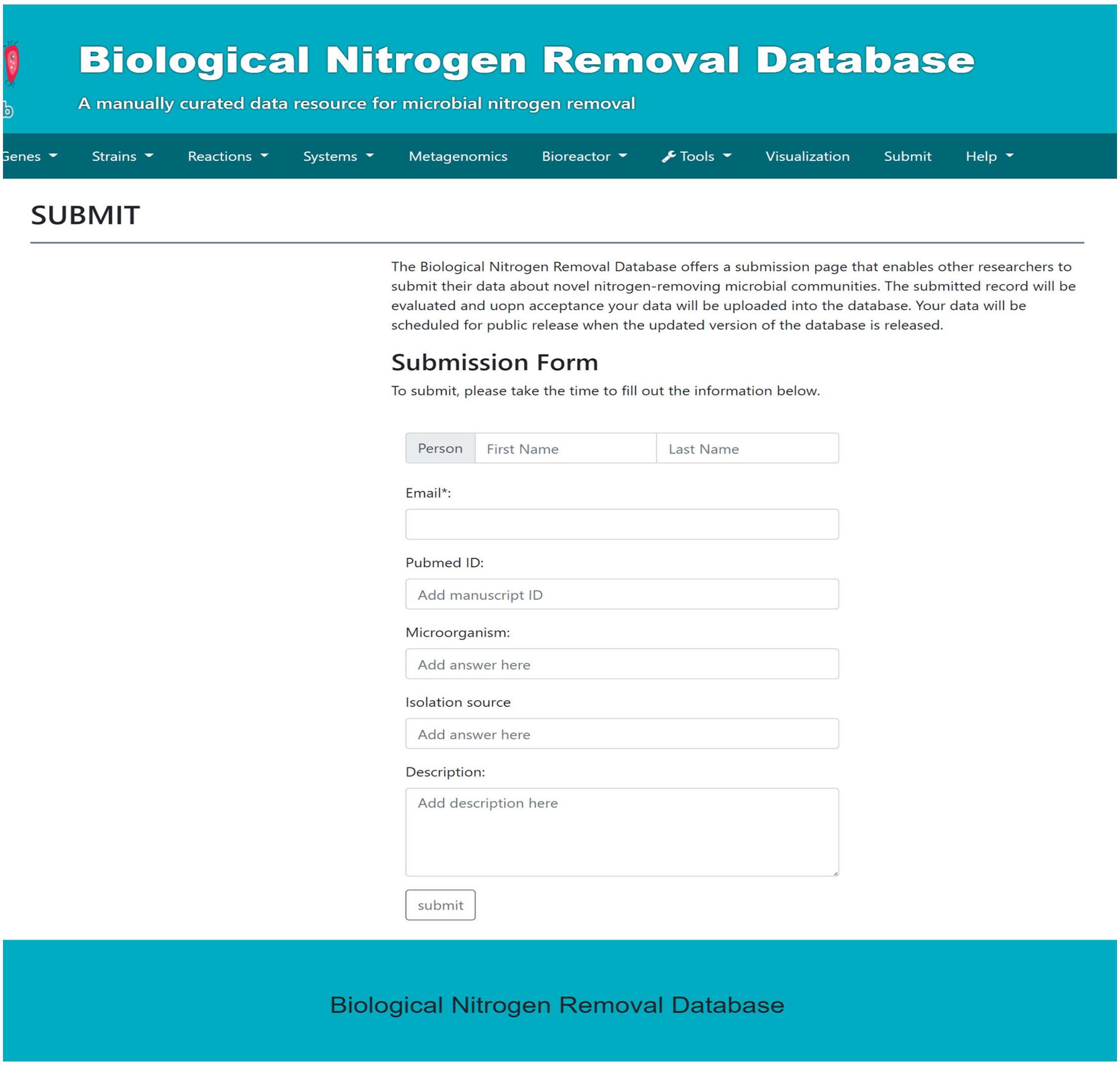

Figure S6. Screenshot of the submission page. An interactive data submission feature is incorporated into the BNRdb, to generate clean and uniform data sets that can be useful to other users. Contributors who wish to submit new data sets related to BNR, which is not already included in the database, can use this feature following the submission guidelines that have been devised. The administrator/curator may then contact the authors who submitted a new dataset to be the editor of that particular dataset and engage in reviewing any inaccurate or potentially missing data during the validation stage. The curator of the database will then upload the new dataset after its systematic validation.
